# Translation and cultural adaptation of the Functional Assessment of Chronic Illness Therapy – Cervical Dysplasia (FACIT-CD) to evaluate quality of life in women with cervical intraepithelial neoplasia

**DOI:** 10.1590/S1679-45082017AO3910

**Published:** 2017

**Authors:** Cristiane Menezes Sirna Fregnani, José Humberto Tavares Guerreiro Fregnani, Carlos Eduardo Paiva, Eliane Marçon Barroso, Mayara Goulart de Camargos, Audrey Tieko Tsunoda, Adhemar Longatto-Filho, Bianca Sakamoto Ribeiro Paiva

**Affiliations:** 1 Hospital de Câncer de Barretos, Barretos, SP, Brazil.; 2 Faculdade de Ciências Médicas, Santa Casa de São Paulo, São Paulo, SP, Brazil.

**Keywords:** Translating, Adaptation, Cervical intraepithelial neoplasia, Quality of life

## Abstract

**Objective:**

To translate and perform the cultural adaptation of the tool Functional Assessment of Chronic Illness Therapy – Cervical Dysplasia (FACIT-CD) to the Portuguese language.

**Methods:**

A descriptive cross-sectional study, with translation and cultural adaptation of the assessment tool performed according to international guidelines and the Functional Assessment of Chronic Illness Therapy (FACIT) protocol group. It involved eight experts, six from Brazil, one from Portugal and one from the United States. After translation and back-translation of the tool, the semantic analysis process was carried out. We randomly included 20 women aged between 18 and 70 years with altered cervical cytology exam, seen at the Department of Prevention and Gynecologic Oncology - *Hospital de Câncer de Barretos*.

**Results:**

The sample consisted of women with low education level. In the first pre-test, ten women participated and half of them considered the questions CD1, CD2 and CD3 as difficult, because they did not understand the meaning of the term “pelvic area”. The question CD5, “I worry about spreading the infection”, was also considered difficult to understand by five women. After the reconsideration of the expert committee and FACIT group, the second pre-test was performed. At this stage, we concluded that the previously raised understanding problems had been solved.

**Conclusion:**

The translated version of FACIT-CD in universal Portuguese language is equivalent to the original version in English and was easily understood by patients with cervical intraepithelial neoplasia.

## RESUMO

**Objetivo:**

Traduzir e adaptar o instrumento *Functional Assessment of Chronic Illness Therapy – Cervical Dysplasia* (FACIT-CD), para a língua portuguesa.

**Métodos:**

Estudo descritivo, transversal, com metodologia de tradução e adaptação cultural de instrumento de avaliação, realizado por meio de diretrizes internacionais e seguindo o protocolo do grupo *Functional Assessment of Chronic Illness Therapy* (FACIT). Envolveu oito especialistas, sendo seis nativos do Brasil, um de Portugal e um dos Estados Unidos. Ao término do processo de tradução e retrotradução, deu-se início ao processo de análise semântica. Foram incluídas aleatoriamente 20 mulheres entre 18 e 70 anos com exame de citologia cervical alterado, atendidas no Departamento de Prevenção e Ginecologia Oncológica do Hospital de Câncer de Barretos.

**Resultados:**

A amostra foi composta por mulheres com baixa escolaridade. No primeiro pré-teste participaram dez mulheres, sendo que a metade considerou as questões CD1, CD2 e CD3 difíceis por não compreenderem o significado do termo “região pélvica”. A questão CD5, “Estou preocupada em disseminar a infecção”, também foi considerada de difícil entendimento por cinco mulheres. Após as reconsiderações do comitê de especialistas e do grupo FACIT, foi realizado o segundo pré-teste. Nesta fase, pode-se concluir que os problemas de entendimento anteriores foram resolvidos.

**Conclusão:**

A versão traduzida do FACIT-CD é equivalente à versão original em inglês e em língua portuguesa universal, sendo facilmente compreendida pelas pacientes com neoplasia intraepitelial cervical.

## INTRODUCTION

Today the World Health Organization estimates there are worldwide approximately 440 million individuals with genital infection by the human papillomavirus (HPV), and roughly 10% of women will carry HPV at some time during their lives.^1,2^ Prevalence of the infection varies from 20 to 40%, depending on the age range and the immunocompetence status; it is more common in young adults.^3^

Many HPV infections are asymptomatic, and in half of the cases, the viruses are eliminated in up to eight months by the immune system. Within 24 months, 90% of women are free of the HPV infection. However, persistent infections lasting longer than 12 months are associated with an increased risk of genital tract cancer.^4^

Despite cervical intraepithelial neoplasias (CIN) being frequently asymptomatic and presenting with a high potential for cure, their diagnosis may bring consequences to the woman, especially in the psychological area, such as a feeling of fear, anxiety, shame, guilt, and reduced self-esteem. There is also the fear related to sexual activity, with reduced number of intercourses and sexual satisfaction.^5^ It is known that HPV infection can cause disorders in the health-related quality of life (HRQOL) of women who carry the virus. Although there are various instruments to objectively evaluate quality of life, few investigate the impact of the CIN on women’s quality of life.

In 2010, Rao et al., published an instrument called Functional Assessment of Chronic Illness Therapy – Cervical Dysplasia (FACIT-CD), which was designed for physical and psychological functional assessment of women with CIN (previously known as “cervical dysplasia”). The instrument was developed in English, and the evaluation of its psychometric properties has not yet been evaluated.^5^

The health evaluation tools of the Functional Assessment of Chronic Illness Therapy (FACIT) system are considered easily administered (self-administered or administered by the interviewer), require a short time for completion, and in general, display good validity and sensitivity for detecting changes.^6^

## OBJECTIVE

To translate and adapt the Functional Assessment of Chronic Illness Therapy – Cervical Dysplasia (FACIT-CD) instrument to Portuguese.

## METHODS

A descriptive cross-sectional study, with a method of translation and cultural adaptation of the assessment instrument, performed by international guidelines and according to the FACIT group protocol.^7-10^

FACIT-CD is a specific instrument for the evaluation of HRQOL in women diagnosed with CIN, composed of 37 questions divided into five scales that measure aspects related to physical well-being (9 questions), treatment satisfaction (4 questions), general perceptions (7 questions), emotional well-being (11 questions), and relationships (6 questions). The questionnaire should be answered taking into consideration the experiences over the last seven days. The response scale is Likert-type, with scores varying between zero and four (from not at all to very much). To calculate the scores, a special manual provided by the FACIT organization, in which a score is attributed to each scale, and then added up to obtain a single value. The total score of the questionnaire can vary from zero to 136. A higher score indicates a better HRQOL.

The translation and adaptation process had two phases. In the first, the translation and adaptation were made. In the second phase, a pre-test was conducted with the objective of certifying comprehension, doubts, and embarrassments of the women interviewed regarding the items and responses of the questionnaires.

The translation and transcultural adaptation of the instrument to Portuguese were performed in accordance with the special form of the FACIT organization and involved eight experts, six from Brazilians, one from Portugal, and one from the United States. To conduct the study, written authorization was obtained from the FACIT organization, as well as prior approval from the Research Ethics Committee of the *Hospital de Câncer de Barretos* (protocol 191,340/2013), CAAE: 12561813.1.0000.5437.

A data collection instrument, when used in another country and/or different culture from that of the original, should follow a rigid method to attain equivalence between the original source and the target language.^7^ There are basically five phases to be traversed, and the first is the translation process, in which at least two bilingual independent individuals are indicated. After translating the instrument, they should meet for a synthesis and consensus between the translations. The version translated to the target language then goes through the back-translation process, returning to the original language. This aims to verify if the translated version reflects the original instrument. The next step is the evaluation by a group of experts, who have the objective of consolidating a pre-final version of the questionnaire. The final phase of the adaptation process is the pre-test, when the instrument is applied to a few people with the purpose of evaluating the level of understanding of the items and responses, thus assuring the equivalence between the versions.^7,8^

**Figure 1:**
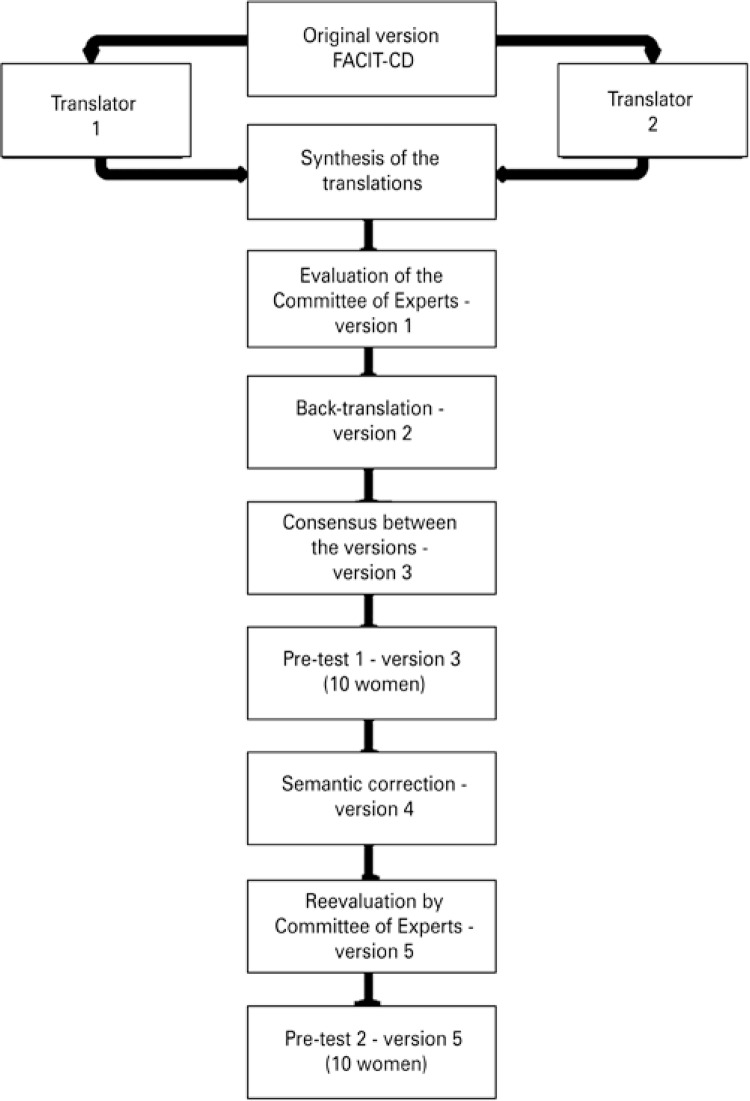
Methodological trajectory of the process of translation and cultural adaptation of the instrument, Functional Assessment of Chronic Illness Therapy – Cervical Dysplasia

The process of translation from English into Portuguese (Figure 1) was carried out by two translators with background in health sciences (a physician and a biologist), one of them from Brazil and one from Portugal, since the objective was translation to universal Portuguese. The translations were done independently (versions 1A and 1B), and then a single version was synthesized (version 1) by three Brazilian researchers (two physicians and one nurse) in a consensus meeting. None of the physicians that participated in the conciliation process translated the tool. Version 1 was back-translated into English by a native translator living in the United States who mastered Portuguese, thus generating version 2. The original version of the instrument and version 2 were compared by two Brazilian observers, who also mastered English and did not participate in the previous steps. Version 3 was obtained by a consensus of these reviewers.

FACIT-CD: Functional Assessment of Chronic Illness Therapy – Cervical Dysplasia.

After the translation process of the FACIT-CD instrument was finalized, according to the method described, the process of semantic analysis began by means of interviews, which had the objective of evaluating relevance, clarity, and comprehension of the version translated into Portuguese. For each question, the understanding level was investigated, as well as the existence of difficult to understand words. During this phase, two pre-tests were performed, each with ten women. In the first one, version 3 of the instrument was used, and localized semantic problems were identified. Corrections generated version 4, which was reevaluated by the expert committee and thus, version 5 was created, which was applied in the second pre-test.

The size of the pre-test sample was empirically defined and followed the method recommended by FACIT.

Twenty women aged 18-70 years were randomly enrolled, and agreed to participate in the research by signing the Informed Consent Form (ICF). Illiterate women or those who were known to have psychological or psychiatric disorders that could prevent them from answering the questionnaires were excluded. The women were recruited at the Department of Prevention and Gynecologic Oncology of the *Hospital de Câncer de Barretos*, and had a prior diagnosis of alterations in the cervical cytology test.

The data were evaluated by descriptive statistics using the software Statistical Package for the Social Sciences (SPSS) version 21.

## RESULTS

Characterization of the populations of pre-tests 1 and 2 is displayed on table 1. The mean age of those interviewed in the first pre-test was 36.9 years, age range 23-60 years. In the second pre-test, the mean age was 44.5 years, and age range 25-63 years. Most women (approximately 45% of sample) reported low schooling level, with incomplete Junior School.

**Table 1:** Sociodemographic and clinical characteristics of the study population (n=20)

Variable	Pre-test 1 (n=10)n (%)	Pre-test 2 (n=10) n (%)
Age, mean (SD=12.61)	36.9	44.5
Schooling		
Incomplete Junior School	5 (50)	4 (40)
Complete Junior School	1 (10)	2 (20)
Incomplete High School	2 (20)	1 (10)
Complete High School	0 (0)	1 (10)
Complete College	2 (20)	2 (20)
Diagnosis		
Cervical intraepithelial neoplasia l	1 (10)	4 (40)
Cervical intraepithelial neoplasia II	4 (40)	3 (30)
Cervical intraepithelial neoplasia III	5 (50)	2 (20)
Invasive carcinoma	0 (0)	1 (10)
Time of diagnosis, years		
>1	9 (90)	1 (10)
<1	1 (10)	9 (90)
Under treatment		
Yes	9 (90)	7 (70)
No	1 (10)	3 (30)
SD: standard deviation.		

Version 3 of the instrument is shown on chart 1. No question was considered irrelevant by the participating women. Questions CD1, CD2, and CD3 were considered difficult by five women, who reported not having understood the meaning of the term “pelvic area” (“pelvic area”). After the considerations of the experts committee and of FACIT, a short explanation was included of the meaning of the pelvic region, adding in parenthesis the term “the lowest part of the belly”. Question CD5, “I worry about spreading the infection” (“I worry about spreading the infection”), was also considered hard to understand by five women, especially because they did not understand the meaning of the term “spreading.” The committee of experts and FACIT chose to add a brief explanation of the term “spreading,” rendering the sentence: “I am concerned about transmitting (spreading to another person) the infection.” Questions CD6, CD7, CD8, CD15, CD16, CD17, CD18, CD19, and CD20 had their text adjusted to facilitate comprehension for Portuguese of Brazil and Portugal, but no new content was added.

**Chart 1:** Composition of the final version of the instrument Functional Assessment of Chronic Illness Therapy – Cervical Dysplasia (FACIT-CD), after translation, back-translation, and review by the experts

Item	Original version (in English)	Version 3 (version utilized in pre-test 1)	Version 4	Version 5 – (version utilized in pre-test 2)
CD1	I have discomfort in my pelvic area	*Tenho desconforto na minha região pélvica*	*Tenho desconforto na minha região pélvica (parte mais baixa da barriga)*	*Tenho desconforto na minha região pélvica (parte mais baixa da barriga)*
CD2	I have pain in my pelvic area	*Tenho dor na minha região pélvica*	*Tenho dor na minha região pélvica (parte mais baixa da barriga)*	*Tenho dor na minha região pélvica (parte mais baixa da barriga)*
CD3	I have cramping in my pelvic area	*Tenho cólicas na minha região pélvica*	*Tenho cólicas na minha região pélvica (parte mais baixa da barriga)*	*Tenho cólicas na minha região pélvica (parte mais baixa da barriga)*
Cx1	I am bothered by discharge or bleeding from my vagina	*O corrimento ou sangramento vaginal incomoda-me*	*O corrimento ou sangramento vaginal incomoda-me*	*O corrimento ou sangramento vaginal incomoda-me*
GP5	I am bothered by side effects of treatment	*Sinto-me incomodada pelos efeitos secundários do tratamento*	*Sinto-me incomodada pelos efeitos secundários do tratamento*	*Sinto-me incomodada pelos efeitos secundários do tratamento*
Q8	Are you sexually active or would you like to be sexually active? If yes, answer the following three questions. If no, skip these questions and move on to the next section	*Você tem vida sexual ativa ou gostaria de ser sexualmente ativa? Se sim, responda as seguintes 3 questões. Se não, pule estas questões e vá para a sessão seguinte*	*Você tem vida sexual ativa ou gostaria de ser sexualmente ativa? Se sim, responda as seguintes 3 questões. Se não, pule estas questões e vá para a sessão seguinte*	*Você tem vida sexual ativa ou gostaria de ser sexualmente ativa? Se sim, responda as seguintes 3 questões. Se não, pule estas questões e vá para a* *seção* *seguinte*
ES8	I have pain or discomfort with intercourse	*Sinto dor ou desconforto durante as relações sexuais*	*Sinto dor ou desconforto durante as relações sexuais*	*Sinto dor ou desconforto durante as relações sexuais*
CD4	I have to limit my sexual activity because of the infection	*Tenho que limitar minha atividade sexual por causa da infecção*	*Tenho que limitar minha atividade sexual por causa da infecção*	*Tenho que limitar* *a* *minha atividade sexual por causa da infecção*
CD5	I worry about spreading the infection	*Estou preocupada em disseminar a infecção*	*Estou preocupada em transmitir (passar para outra pessoa) a infecção*	*Estou preocupada em transmitir (passar para outra pessoa) a infecção*
GR1	I have confidence in my doctor(s)	*Tenho confiança no(s) meu(s) médico(s)*	*Tenho confiança no(s) meu(s) médico(s)*	*Tenho confiança no(s) meu(s) médico(s)*
CD6	I feel that I received the treatment that was right for me	*Sinto que recebi o tratamento que foi correto para mim*	*Sinto que recebi o tratamento correto para mim*	*Sinto que recebi o tratamento correto para mim*
CD7	My doctor gave me explanations that I could understand	*Meu médico me deu explicações que eu consegui compreender*	*O meu médico deu-me explicações que eu consegui compreender*	*O meu médico deu-me explicações que eu consegui compreender*
CD8	My doctor explained the possible benefits of my treatment	*Meu médico me explicou os possíveis benefícios do meu tratamento*	*O meu médico explicou-me os possíveis benefícios do meu tratamento*	*O meu médico explicou-me os possíveis benefícios do meu tratamento*
GF1	I am able to work (include work at home)	*Sou capaz de trabalhar (inclusive em casa)*	*Sou capaz de trabalhar (inclusive em casa)*	*Sou capaz de trabalhar (inclusive em casa)*
GF3	I am able to enjoy life	*Sou capaz de sentir prazer em viver*	*Sou capaz de sentir prazer em viver*	*Sou capaz de sentir prazer em viver*
H11	I am hopeful about the future	*Tenho esperança quanto ao futuro*	*Tenho esperança quanto ao futuro*	*Tenho esperança quanto ao futuro*
Sp9	I find comfort in my faith or spiritual beliefs	*Encontro conforto na minha fé ou crenças espirituais*	*Encontro conforto na minha fé ou crenças espirituais*	*Encontro conforto na minha fé ou crenças espirituais*
GF7	I am content with the quality of my life right now	*Estou satisfeita com a qualidade da minha vida neste momento*	*Estou satisfeita com a qualidade da minha vida neste momento*	*Estou satisfeita com a qualidade da minha vida neste momento*
CD9	I feel that I can manage things that come up around this infection	*Sinto que posso lidar com as coisas que surgem com esta infecção*	*Sinto que posso lidar com as coisas que surgem com esta infecção*	*Sinto que posso lidar com as coisas que surgem com esta infecção*
CD10	I have accepted that I have this infection	*Aceitei que tenho esta infecção*	*Aceitei que tenho esta infecção*	*Aceitei que tenho esta infecção*
CD11	I worry that the infection will get worse	*Estou preocupada que a infecção piore*	*Estou preocupada que a infecção piore*	*Estou preocupada que a infecção piore*
CD12	I have hidden this problem so others will not notice	*Tenho escondido este problema para que os outros não percebam*	*Tenho escondido este problema para que os outros não percebam*	*Tenho escondido este problema para que os outros não percebam*
CD13	I have concerns about my ability to become pregnant	*Estou preocupada sobre a minha capacidade de engravidar*	*Estou preocupada sobre a minha capacidade de engravidar*	*Estou preocupada sobre a minha capacidade de engravidar*
BMT 18	The cost of my treatment is a burden on me or my family	*O custo do meu tratamento é um peso para mim ou para a minha família*	*O custo do meu tratamento é um peso para mim ou para a minha família*	*O custo do meu tratamento é um peso para mim ou para a minha família*
CD14	I worry about other people’s attitudes towards me	*Estou preocupada com as atitudes das outras pessoas em relação a mim*	*Estou preocupada com as atitudes das outras pessoas em relação a mim*	*Estou preocupada com as atitudes das outras pessoas em relação a mim*
CD15	I feel embarrassed about the infection	*Sinto-me envergonhada com a infecção*	*Sinto-me envergonhada por causa da infecção*	*Sinto-me envergonhada por causa da infecção*
CD16	I tend to blame myself for the infection	*Tenho a tendência de me culpar pela infecção*	*Tenho tendência para me culpar pela infecção*	*Tenho tendência para me culpar pela infecção*
CD17	I was careful who I told about the infection	*Tive cuidado para quem eu contei sobre a infecção*	*Tive cuidado a quem eu contei sobre a infecção*	*Tive cuidado a quem eu contei sobre a infecção*
CD18	I have had difficulty telling my partner/spouse about the infection	*Tive dificuldade em contar ao meu parceiro/marido sobre a infecção*	*Tive dificuldade em contar para meu parceiro/ marido sobre a infecção*	*Tive dificuldade em contar* *ao* *meu parceiro/marido sobre a infecção*
CD19	I am frustrated by the infection	*Estou frustrada por causa da infecção*	*Estou frustrada pela infecção*	*Estou frustrada* *por causa* *da infecção*
CD20	I am depressed about the infection	*Estou deprimida por causa da infecção*	*Estou deprimida pela infecção*	*Estou deprimida* *por causa* *da infecção*
Q9	I have told my partner/spouse about my infection:	*Contei ao meu parceiro/marido sobre a minha infecção:*	*Contei ao meu parceiro/marido sobre a minha infecção:*	*Contei ao meu parceiro/marido sobre a minha infecção:*
	No ___ Yes___If yes:	*Não ____ Sim____ Se sim:*	*Não ____ Sim____ Se sim:*	*Não ____ Sim____ Se sim:*
CD21	I get emotional support from my partner/spouse	*Recebo apoio emocional do meu parceiro/marido*	*Recebo apoio emocional do meu parceiro/marido*	*Recebo apoio emocional do meu parceiro/marido*
Q10	I have told family members about my infection:	*Contei aos meus familiares sobre a minha infecção:*	*Contei aos meus familiares sobre a minha infecção:*	*Contei aos meus familiares sobre a minha infecção:*
	No ___ Yes___If yes:	*Não ____ Sim____ Se sim:*	*Não ____ Sim____ Se sim:*	*Não ____ Sim____ Se sim:*
CD22	I get emotional support from family members	*Recebo apoio emocional dos meus familiares*	*Recebo apoio emocional dos meus familiares*	*Recebo apoio emocional dos meus familiares*
GS1	I feel close to my friends	*Sinto que tenho uma boa relação com os meus amigos*	*Sinto que tenho uma boa relação com os meus amigos*	*Sinto que tenho uma boa relação com os meus amigos*
H13	I have people to help me if I need it	*Tenho pessoas que podem ajudar em caso de necessidade*	*Tenho pessoas que podem ajudar em caso de necessidade*	*Tenho pessoas que podem ajudar em caso de necessidade*

In the fourth version of the FACIT-CD instrument, there were minimal alterations pointed out by the committee of experts, once again for localized adjustment of the text. In question Q8, the word “*sessão*” was replaced by “*seção*”; in question CD4, an “*a*” was added for a better interpretation; in CD18, the preposition “*para”* was changed to “*ao”*, and in questions CD19 and CD20, the choice was made to exchange “*pela*” for “*por causa*”. Thus, version 5 of the FACIT-CD instrument was generated.

Version 5, the final one of the instrument, was applied in the second pre-test. During this phase, there were no doubts regarding questions CD1, CD2, CD3, and CD5. Only one woman had difficulty understanding question CD10 (“I have accepted that I have this infection”). The question was analyzed by the specialist committee and since there were no problems with understanding in the first pre-test, it was decided to maintain the wording of the sentence.

## DISCUSSION

Currently, there are many indications that support the benefits of evaluating the HRQOL of women who carry HPV. This infection is considered the most frequent sexually transmitted disease all over the world.^1^ Most cases do not present with symptoms, and the persistence of the infection is related primarily to the development of cervical cancer.^2,11^ The main impact cause by diagnosis of HPV infection is related to social and psychological factors. Since it is a sexually transmitted disease, and with the possibility of developing other HPV-related conditions, it is common for patients to experience anxiety, stress, fear, complications in interpersonal relationships, among other feelings.^12,13^ Information and knowledge about the disease are the main factors to minimize psychological suffering. Therefore, one can emphasize the importance of measuring the HRQOL in women infected by HPV.

In this way, an objective assessment instrument for the physical and psychological functional evaluation of women with CIN can be very useful for clinical practice. The purpose of the present study was to translate and culturally adapt the FACIT-CD instrument to the universal Portuguese language, since until now, in Brazil there is no other instrument developed or considered valid that evaluates the HRQOL of women with CIN.

The entire methodological phase of translation and back-translation was thoroughly complied with, according to the method by Beaton et al., and to the orientations described in the FACIT group protocol, maintaining the consistency between the original and the adapted versions.^7^ It is important to mention that the most significant changes were made in the instrument after the first pre-test. In this phase, the low schooling level might have influenced understanding of some of the questions. The items in the questionnaire containing the expression “pelvic region” and “spread” were not understood by half of the women interviewed. These are terms commonly used by healthcare professionals, but not by the population in general. Especially when the difficulty in understanding of some words was observed in half of the women interviewed in the first phase, we noted that, with the changes made and the additional explanations, performing the second pre-test occurred without further difficulties in understanding the questions that were restructured. It is important to remember that, according to the Brazilian Institute of Geography and Statistics (IBGE - *Instituto Brasileiro de Geografia e Estatística*), Brazil is a highly populated country (approximately 200 million inhabitants), with important socioeconomic issues and problems related to access to education. Half of the population (49.25%) over 25 years of age has not completed junior school (9 years of education), which represents 54.5 million people in this age group; 8.6% of the population aged over 15 years is illiterate; and another 33 million Brazilians (about 18%) are considered functionally illiterate, that is, they have completed less than four years of schooling.^14^

In this study, 45% of women reported low schooling level (incomplete junior school). However, the final version of the instrument could be understood by women, which leads one to think that the questionnaire may be applied with relative ease within the Brazilian context. Other Brazilian studies have pointed out the low education level as not being a significant factor in the translation and validation of quality of life instruments.^15,16^

Even though the objective of this study was to translate the FACIT-CD instrument to universal Portuguese, a semantic and cultural adaptation is needed in other countries where the official language is Portuguese, such as, for example, Portugal, Mozambique, Angola, Cape Verde, and East Timor, among others.

It is necessary to point out that women affected by CIN generally present with a greater number of psychological complaints, and the FACIT-CD instrument is composed of multiple questions that cover the theme, besides addressing other important issues, such as physical well-being, treatment satisfaction, and even questions that include the evaluation of relationships.

Although it was possible to translate the instrument into Portuguese, it is still necessary to evaluate its psychometric aspects in other studies before it is used to assess quality of life in women with CIN, both in clinical practice and in further studies.

The process of evaluation of the psychometric properties of the translated and adapted version of FACIT-CD is still ongoing by the main author of this article.

## CONCLUSION

This study demonstrated that the version of FACIT-CD translated into Portuguese is equivalent (semantically, conceptually, and culturally) to the original version in English and it is easily understood by patients with cervical intraepithelial neoplasia. Therefore, it was considered suitable for the validation phase, which is currently underway.
