# *Erratum:* Translation and cultural adaptation of the Functional Assessment of Chronic Illness Therapy – Cervical Dysplasia (FACIT-CD) to evaluate quality of life in women with cervical intraepithelial neoplasia

**DOI:** 10.1590/S1679-45082017AO3910E

**Published:** 2017

Cristiane Menezes Sirna Fregnani ^1^ ,

José Humberto Tavares Guerreiro Fregnani ^1^ ,

Carlos Eduardo Paiva ^1^ ,

Eliane Marçon Barroso ^1^ ,

Mayara Goulart de Camargos ^1^ ,

Audrey Tieko Tsunoda ^1^ ,

Adhemar Longatto-Filho ^1^ ,

Bianca Sakamoto Ribeiro Paiva ^1^

^1^ Hospital de Câncer de Barretos, Barretos, SP, Brazil.

In the manuscript “Translation and cultural adaptation of the Functional Assessment of Chronic Illness Therapy – Cervical Dysplasia (FACIT-CD) to evaluate quality of life in women with cervical intraepithelial neoplasia”, DOI number 10.1590/S1679-45082017AO3910, published at einstein (São Paulo). 2017;15(2):155-61, on page 161, the institutional affiliation for José Humberto Tavares Guerreiro Fregnani has been changed. The authors regret this error.

**Stated**: 

^2^ Faculdade de Ciências Médicas, Santa Casa de São Paulo, São Paulo, SP, Brazil.

**It should be read:**

^1^ Hospital de Câncer de Barretos, Barretos, SP, Brazil.

